# Hæmatemesis

**Published:** 1869-06

**Authors:** F. K. Bailey

**Affiliations:** Knoxville, Tennessee


ARTICLE XXII.

### HÆMATEMESIS.

By F. K. BAILEY, M.D., Knoxville, Tennessee.

I was called, November 18th, 1868, to see a lady, aged 52, a native of one of the New England States, but for 30 years resident of a malarious district in Ohio. For 35 years she had suffered from a painful affection of the stomach, which was called by her physicians dyspepsia. During the last 10 years, she had been subject to chills, attended with great depression, palpitation, fainting, and other symptoms indicating shock to the vital powers. In October, she came to this place, her medical attendant having advised change of climate, and who also expressed his opinion that she could not survive another Northern winter. She endured the journey without much fatigue, and finding herself very pleasantly situated at the home of her son, as well as feeling that the mildness of our autumn air might prove beneficial, was greatly encouraged with the prospect of improving health.

November 16th. She walked about half a mile, and returned feeling somewhat fatigued. The next day she felt symptoms of a chill, such as she had experienced for a long time after overexertion, and took some quinine.

During the forenoon of the 18th, she became suddenly faint, and very distressingly sick at the stomach. In a few minutes, she vomited more than a pint of partially coagulated blood.

I was soon called, and saw her before reaction was established. I found the blood she had vomited was unchanged in color, and it appeared as though it had flowed very rapidly from an artery. She was deathly pale; pulse 130, and very feeble. Not able to speak so as to give any statement of her feelings. A member of the family had given her some common salt, before my arrival, and she had not thrown up any blood after it was administered.

I gave her two grains of acetate of lead, and six grains of Dover's powder; applied sinapisms to the pit of the stomach and the feet. In about 15 minutes after my arrival at the bedside, she threw up four or five ounces of dark blood. Reaction came up very slowly, and it was nearly dark before she was able to converse sufficiently to give an account of her feelings, or a history of her case. Having never seen her before, I could learn nothing from her, except what she was able to tell while still very feeble.

Judging from her paleness of countenance, and the fact of a residence in a malarious district, I suspected congestion of some of the abdominal viscera. To obviate a possible recurrence, after an interval of the usual length of time in intermittents, I gave sulphate of quinine, in 5-grain doses, to be repeated every three hours. Also ordered warm toddy to be given occasionally during the night, and to keep the extremities warm.

Thursday, 19th: *8 A.M.* My patient has slept but little during the night, was very restless, and suffered extremely from nausea and general depression. Pulse 140, small and feeble.

No vomiting; but still complains of severe distress and nausea. To continue quinine in lesser doses, and to give persulphate of iron in solution, every hour.

*7 P.M.* Still very restless, and much depressed. Pulse 140, very small, and slightly wiry. Tenderness on pressure over the region of the stomach, and as low as the umbilicus. Indications of peritoneal inflammation in the small wiry pulse, and a redness of the tongue.

Prescribed bromide potassa, in 15-grain doses, every three hours. Small doses sulph. quinine, and stimulation, if the pulse should become more feeble.

Friday, 20th: *7 A.M.* My patient had slept four or five hours during the night, and expressed herself as feeling much better. No vomiting, nor has there been any alvine evacuation since the attack. No abdominal distention. Great thirst, with some nausea. Pulse 140, and still very small. To take mucilage gum Arabic, with eight drops oil turpentine, every three hours, and toddy *pro re nata.*

*7 P.M.* No improvement in general appearance. There has been one evacuation from the bowels of a very dark grumous appearance, evidently changed blood. To continue mucilage and stimulants.

Saturday, 21st: *8 A.M.* Patient has slept but little during the night. Pulse very small, and 150. No vomiting. To take toddy and beef-tea. Was called at noon, and found she had vomited a half pint of blood, coagulated, and very dark, and voided, at the same time, a quantity of very black grumous substance from the bowels. Very much exhausted. Pulse 160, and hardly perceptible.

Continued to sink, and expired at midnight.

There was no *post mortem* examination.

The above case seems to be one of a class, in which a person will suffer from a painful affection of the stomach for years, and will be treated for gastrodynia, or neuralgia. The mere fact of vomiting blood throws but little light upon this case; for although the blood came from the stomach, we do not know how it came to be in that cavity. From the fact of the blood vomited being fresh, together with the quantity, would indicate that it escaped from no small source. If it had escaped slowly from the mucous membrane of the stomach, the color would have been changed.

Remarks illustrative of this are found in No. 57, page 113.

Writers speak of cases in which blood will escape from a vessel denuded in a malignant tumor, which may have been forming for years, and suddenly ruptured.

There seemed to me some indications of inflammation of a low grade, following immediately upon the escape of the blood, shown by the small and very frequent pulse.

I have known, at different times in my practise, cases in which a person will have occasional attacks of vomiting pus, mixed with blood, which at last terminated fatally.

The diagnosis in those cases was, an ulcer, which would at intervals pour out its contents into the duodenum, or stomach, to be rejected by vomiting, and also accompanied with a profuse quantity of the same matter, evacuated from the rectum. I think I never before met with *such* a case as the one above described.

Vomiting of blood is often met with from vicarious menstruation, and local congestion of the gastric mucous membrane, which may be easily relieved.

Some 20 years ago, I had a case of a simple quotidian intermittent, in which the patient, a very large and fat man, vomited blood very freely during the hot stage. The blood ejected was fresh, and not coagulated in the least.

He had a second or third recurrence of paroxysms before anti-periodics could be made to suspend the disease; and the vomiting of blood invariably returned with the hot stage. I will add that the subject was a person addicted to excessive use of intoxicating drinks, and as a congestion of the mucous lining of the stomach is known to exist in such cases, the escape of blood is easily accounted for in the hot stage of fever.

Hæmatemesis is, by some, placed in the list of diseases of the stomach; but it can only be considered as a result of some other morbid condition. Vomiting of blood should no more be called a distinct disease than vomiting of bile; and when we find a person throwing from the stomach a quantity of blood, the cause of its presence in that cavity must be sought, and means adopted for its removal.

